# Near Miss Reporting and Organizational Learning in Health Care: Conceptual Framework Development Study

**DOI:** 10.2196/87846

**Published:** 2026-04-22

**Authors:** Mohammed As'ad

**Affiliations:** 1 Dr Sulaiman Al Habib Medical Group, Corporate QPS, Headquarters, Al Awsat Valley S, Al Olaya, Riyadh, 12241, Saudi Arabia, 966 11525999

**Keywords:** near miss, patient safety, statistical process control, safety culture, quality improvement, acute care, hospital

## Abstract

**Background:**

Near miss events can reveal system problems before patients are harmed, but current reviews are inconsistent and often rely on simple counts that are distorted by patient volume and reporting culture. Consequently, leaders cannot tell whether a rise in reports means that safety is getting worse or that staff are reporting more, and current systems are not strong enough to clearly separate real safety risks from random variation.

**Objective:**

This study developed a 3-level near miss framework (NM³), a conceptual framework that converts descriptive near miss data into decision-grade intelligence through a structured, evidence-based process, including baseline measurement and advanced interpretation and governance.

**Methods:**

NM³ was developed to provide decision-grade analytics for acute inpatient hospital settings. The framework was designed as a maturity model, progressing from baseline measurement to advanced interpretation. It integrates standardized definitions, rate calculations, statistical process control, severity weighting, and learning metrics.

**Results:**

Level 1 establishes an organizational baseline through near miss rates per 1000 patient-days and near miss–to-harm ratios monitored with control charts. Level 2 introduces domain-specific denominators and unit-level charts to detect local variation. Level 3 applies severity weighting to generate a Near Miss Index; incorporates learning yields at 90 and 180 days; and triangulates near miss trends with harm events, exposure, reporting volume, and culture measures. A synthetic example demonstrates how the framework converts raw reports into stable rates, weighted indices, and learning metrics.

**Conclusions:**

NM³ provides a structured pathway for organizations to strengthen near miss analytics. By progressing through maturity levels, leaders can improve the interpretation of safety signals, prioritize high-consequence risks, and integrate near miss reporting into governance.

## Introduction

### Background

Near miss events, which reveal systemic hazards before harm occurs, are fundamental to proactive safety governance. Global health policies now emphasize their systematic analysis, a principle operationalized in frameworks such as the National Health Service England’s Patient Safety Incident Response Framework [1-3]. Consequently, leaders must treat near miss data as a crucial leading indicator of systemic safety rather than as a secondary metric [1].

Effective safety oversight requires analyzing trends rather than isolated event counts. Best practices advocate interpreting safety indicators (eg, rates of near misses, medication errors, patient falls, or infection events) alongside workforce culture and patient experience metrics to discern meaningful signals from statistical noise, thereby strengthening governance [4,5]. However, translating this into practice is fraught with methodological challenges. Voluntary reporting systems suffer from significant underascertainment due to reporting culture, workload, and fear [6,7]. These barriers disproportionately suppress near miss submissions compared to harm incidents, distorting data and undermining valid comparisons [8-10].

Analytical choices add further uncertainty. Raw event counts are confounded by patient volume and reporting behavior, while meaningful rates require domain-aligned denominators [4]. Furthermore, overdispersion is common in health care data, meaning that conventional statistical process control (SPC) charts can produce false signals if used without adjustment [11]. Although corrective methods such as the Laney p’ chart exist [12], they have not been embedded within a structured near miss analytic framework that links SPC to event classification, severity weighting, and organizational learning—a gap this study addresses.

### Objectives

These challenges create a critical interpretive dilemma for leaders: does a rise in reported near misses signify deteriorating safety or an improving reporting culture? Without standardized terminology, risk-adjusted denominators, and dispersion-robust control charts, leaders risk misinterpreting these vital data, misallocating resources, and failing to address systemic vulnerabilities [1,4,13,14].

From a human factors perspective, this interpretive dilemma reflects a classic problem of decision-making under uncertainty within complex sociotechnical systems [15]. Leaders must derive meaning from incomplete, socially mediated, and variably coded safety signals. In the absence of structured analytic support, interpretation depends on informal sensemaking [16] and locally constructed mental models [17], increasing the risk of inconsistent prioritization and resource allocation.

Therefore, the objective of this study was to develop a conceptual framework that provides a structured, evidence-based methodology for near miss analysis. To achieve this, we designed a 3-level maturity model that integrates standardized terminology, SPC, severity weighting, and novel learning metrics. This structured approach aims to provide organizational leaders with reliable, decision-grade intelligence to guide and sustain safety improvement initiatives. The 3-level near miss framework (NM³) does not treat reporting frequency as a safety outcome; rather, it provides a structured analytic pathway that transforms near miss data into interpretable, learning-oriented intelligence for governance and improvement.

## Methods

### Design and Approach

This study reports the development of a conceptual framework for analyzing near miss safety events, following the integrative methodology by Jabareen [18]. This method proceeds through a series of iterative phases: mapping selected data sources, reading and categorizing the data, identifying and naming concepts, deconstructing and categorizing those concepts, integrating them into a coherent framework, and validating the result through an iterative process of rethinking and synthesis. The approach constructs interrelated concepts by synthesizing theory and evidence rather than through empirical data collection. The aim was to create a structured, 3-level model for near miss analytics that offers decision-grade intelligence to support organizational safety governance. The process was exclusively analytic and integrative and did not involve human participants.

### Evidence and Theoretical Sources

The framework integrates principles from peer-reviewed research and authoritative institutional guidance. To align with established international standards, its foundational definitions were derived from the World Health Organization (WHO) International Classification for Patient Safety (ICPS) and Agency for Healthcare Research and Quality (AHRQ) Common Formats for Event Reporting–Hospital (version 2.0) [19,20]. The analytical components were informed by literature on the application of SPC in health care, including tutorials on chart construction and reviews of its effectiveness [20-22]. Methods for weighting event severity were adapted from the Institute for Healthcare Improvement (IHI) Severity Assessment Code (SAC) Matrix. This tool is widely used in root cause analysis [23]. To account for known limitations in reporting data, the design was informed by recent systematic reviews that identify common barriers to near miss reporting [6,8]. Conclusively, to ensure contemporary policy relevance, the framework incorporates strategic priorities for safety measurement from key governance documents issued by the WHO and National Health Service England [1,3,19]. Only peer-reviewed publications and official institutional reports were used in the synthesis.

### Framework Construction

#### Overview

The framework was constructed as a staged maturity model with 3 progressive levels, each representing greater analytic depth for interpreting near miss data. Construction followed a sequence of definitional alignment, operational specification, and integration into maturity levels. International taxonomies provided the definitional base, operational rules were derived from methodological literature on SPC and risk assessment, and maturity staging was informed by organizational safety models that emphasize progressive capacity building [18,19,24]. The 3 levels are summarized in Table 1, which presents their scope, core components, and associated formulas. These definitions and operational rules are further detailed and operationalized in Table 2 to ensure direct alignment between theoretical sources and the applied framework design. Together, these tables codify the design specifications of the framework and ensure reproducibility in application.

**Table 1.** Summary of the 3 levels of the decision-grade near miss framework^a^.

Level	Scope and purpose	Core components	Formulas and calculations
Level 1—essential minimum (organization-wide baseline)	Establish consistent, decision-usable metrics across the whole hospital system	Standardizes definitions (World Health Organization or Agency for Healthcare Research and Quality taxonomy: near miss, no-harm incident, unsafe condition, and incident) Records if the event reached the patient Captures detection mode (human, alarm, patient, and chance) Collects system-wide numerators (counts) Uses a single denominator: patient-days or admissions Applies SPC^b^ (u-chart) for rates	Near miss rate=(near misses÷patient-days)×1000 Reported near miss–to-harm ratio=reported near misses÷reported harm events SPC=u-chart (Laney U’ if overdispersed)
Level 2—targeted stratification (high-risk or high-volume areas)	Enables hot spot detection in specific clinical units or processes	Applies domain-specific denominators ICU^c^ or wards: per 1000 patient-days Pharmacy: per 10,000 doses Laboratory: per 1000 specimens Transfusion: per 1000 units Procedures: per 1000 surgeries or diagnostic studies Generates unit-level SPC charts (interpreted trend over time rather than as league tables) Identifies clusters and recurrent themes	Domain near miss rate=(near misses÷domain exposures)×constant Examples ICU: (near misses÷ICU patient-days)×1000. Pharmacy: (near misses÷doses dispensed)×10,000 Unit-level SPC: separate u-charts by ward or domain
Level 3—advanced decision-grade analytics (mature organizations)	Prioritizes risks by consequence and integrates cultural context for leadership decision-making	Applies IHI SAC^d^ scoring Creates a severity-weighted NMI^e^ Tracks corrective action yield Percentage implemented by 90 days Percentage sustained at 180 days Triangulates with harm events, reporting volumes, exposure, and safety culture survey results	Severity-weighted NMI=Σ(IHI SAC scoring×event count) Learning yield (90 days)=actions implemented÷near misses Learning yield (180 days)=actions sustained÷actions implemented Triangulation=interpretation of NMI, rates, harm events, and culture indicators together

a This table presents the staged maturity model used to interpret near miss data. Level 1 (essential minimum) provides an organization-wide baseline using standardized taxonomy, patient-day denominators, near miss rates, near miss–to-harm ratios, and statistical process control with Laney U’ adjustment when overdispersion occurs. Level 2 (targeted stratification) adds diagnostic resolution with domain-specific denominators such as intensive care unit patient-days, medication doses, laboratory specimens, transfusion units, or procedures and generates unit-level u-charts interpreted longitudinally. Level 3 (advanced decision-grade analytics) introduces Institute for Healthcare Improvement Severity Assessment Code scoring; a Near Miss Index; learning yield metrics at 90 and 180 days; and triangulation with harm rates, exposure, reporting volume, and safety culture survey data.

b SPC: statistical process control.

c ICU: intensive care unit.

d IHI SAC: Institute for Healthcare Improvement Severity Assessment Code.

e NMI: Near Miss Index.

**Table 2.** Data elements and operational definitions used across the 3 levels of the near miss framework^a^.

Data element	Operational definition	Allowed values or codes	Levels used	Source	Notes or pitfalls
Event type	Classification of an incident according to the World Health Organization or the Agency for Healthcare Research and Quality taxonomy	Near miss, no-harm incident, and harmful incident	L1, L2, and L3	[20,25]	Risk of misclassification if definitions are unclear
Reached patient	An indicator of whether the event reached the patient	Yes or no	L1, L2, and L3	[25]	Must be clearly distinguished from “no-harm incident”
Detection mode	How the event was detected	Human, alarm, patient, and chance	L1, L2, and L3	[25]	Requires training for consistent classification
Unit or domain	Location or process domain in which the event occurred	Intensive care unit, operating room, emergency department, pharmacy, laboratory, and transfusion	L2 and L3	Adapted from quality indicator conventions	Essential for stratification
Denominator type	Exposure measure aligned to the domain	Patient-days, doses, specimens, units, and procedures	L1 and L2	[21,26]	Denominator accuracy depends on data systems
Near miss rate	Standardized rate of near misses	(Near misses÷denominator)×scaling constant	L1 and L2	[21,26]	Must aggregate if denominators are too small
Near miss–to-harm ratio	Ratio of near misses to harmful incidents	Integer ratio	L1	[18,19,24]	Requires reliable coding of harm incidents
Severity score	Potential consequences if the event had not been intercepted	Minor, moderate, major, and catastrophic	L3	[23]	Interrater agreement is needed
Near Miss Index	Aggregate severity-weighted index of near misses	Σ(Institute for Healthcare Improvement Severity Assessment Code×event count)	L3	[23]	Sensitive to scoring reliability
Learning yield (90 days)	Proportion of near misses with actions implemented within 90 days	Action implemented÷near misses	L3	[27]	Requires reliable action tracking
Learning yield (180 days)	Proportion of implemented actions sustained at 180 days	Actions sustained÷actions implemented	L3	[28]	Measurement of sustainability is often weak
Triangulation indicators	Complementary safety indicators for context	Harm rates, reporting volume, and culture scores	L3	[29,30]	Must avoid simplistic correlations

a This table defines the data elements required for consistent measurement and interpretation of near miss events. It includes classifications, denominators, severity scoring, and learning metrics, with allowed values, levels of use, sources, and potential pitfalls. L1 (level 1: essential minimum) refers to organization-wide baseline measurement of near misses using standardized taxonomy, patient-day denominators, rates, and statistical process control (SPC) monitoring. L2 (level 2: targeted stratification) applies domain-specific denominators (eg, intensive care unit, pharmacy, laboratory, transfusion, and procedures) and unit-level SPC charts for hot spot detection. L3 (level 3: advanced decision-grade analytics) incorporates severity scoring; the Near Miss Index; learning yields at 90 and 180 days; and triangulation with harm, exposure, reporting volume, and culture indicators.

#### Level 1: Essential Minimum

The first level establishes a standardized foundation for organization-wide surveillance of near miss events. At this stage, events are classified using the WHO ICPS and the AHRQ’s Common Formats, which define the categories of near miss, no-harm incident, and harmful incident [20,25]. Each event is recorded with an indication of whether it reached the patient and with specification of the detection mode, classified as human intervention, automated alarm, patient self-report, or chance discovery. These data elements are defined in Table 2, which provides the operational specifications required at all 3 levels.

For rate construction, the denominator is hospital patient-days, consistent with conventions used for monitoring hospital-acquired conditions such as falls and infections [26]. The calculations specified for this level, summarized in Table 1, are the near miss rate, defined as the number of near misses per 1000 patient-days, and the reported near miss–to-harm ratio, defined as the number of reported near misses divided by the number of reported harmful incidents. Temporal analysis is performed using u-charts, which accommodate variable denominators, and the Laney U’ modification is applied when overdispersion is identified, ensuring stable control limits and valid interpretation of signals [21,26,31].

#### Level 2: Targeted Stratification

The second level adds analytic depth by applying stratification to high-risk or high-volume domains in which the opportunity for near misses is greatest. The framework specifies denominators that align with domain-specific exposures (Table 1): for intensive care, near misses per 1000 intensive care unit patient-days; in pharmacy services, near misses per 10,000 medication doses; in laboratory settings, near misses per 1000 specimens; in transfusion services, near misses per 1000 blood units; and in procedural areas, near misses per 1000 surgeries or diagnostic studies. These denominators are drawn from existing quality indicator conventions and are further defined in Table 2 to ensure consistent application across settings. Organizations must ensure these definitions are applied with high fidelity, as variations in how they are operationalized can compromise consistency and comparability. For example, a “procedure” or a “dispensed dose” can introduce measurement error.

Each domain generates unit-level u-charts that are interpreted longitudinally within the unit rather than across units, reflecting best practice in the application of SPC for quality improvement [21,26]. Where denominators fall below stability thresholds, data are aggregated to quarterly intervals to reduce small-number instability, which is a recognized limitation in attribute charting for health care data [26]. Aggregation applies primarily to low-volume domains such as transfusion or pharmacy services; higher-volume domains (eg, intensive care units or surgical units) maintain monthly analysis to preserve sensitivity to special-cause variation.

#### Level 3: Advanced Decision-Grade Analytics

Level 3 introduces prioritization and learning metrics. Severity weighting was adapted from the IHI SAC Matrix [23]. Each near miss is scored on a 4-point ordinal scale representing the worst reasonable outcome if not intercepted: minor, moderate, major, or catastrophic. The Near Miss Index (NMI) is calculated by summing severity scores across events in a reporting period. To capture the organizational response, specific learning metrics were defined. However, systematic reviews indicate that only a minority of reported incidents lead to implemented systemic changes, highlighting a critical gap between reporting and learning [27].

*Learning yield (90 days*) measures the proportion of near misses that generate an implemented corrective action within 90 days of reporting. This time frame is critical for ensuring timeliness; health care literature shows that prolonged delays between incident reporting and visible intervention can erode frontline staff engagement and undermine trust in the safety system [32]. *Learning yield (180 days*) assesses the proportion of those initial actions that are sustained at the 180-day mark. This follow-up period is a standard in implementation science used to differentiate a temporary, reactive solution from a change that has been successfully embedded into routine clinical practice. Evidence of sustainment should be determined through objective methods such as process audits or analysis of postimplementation performance data [28]. The 90- and 180-day intervals are operational thresholds specified by this framework to operationalize responsiveness and sustainment. Therefore, these metrics align with quality improvement literature by providing quantitative indicators of a functional learning system, measuring both its immediate responsiveness (action closure) and its ability to create lasting change (sustainment) [33]. Importantly, learning yield is a process metric that quantifies the timeliness and persistence of organizational response; it does not measure whether the implemented actions were effective at reducing harm. Demonstrating intervention effectiveness would require outcome-level studies with appropriate controls, which lies beyond the scope of a reporting-based analytic framework. Therefore, learning yield should be interpreted as evidence of a functioning learning loop rather than proof of harm reduction.

Finally, triangulation was embedded to contextualize near miss data. Framework design requires that near miss signals be interpreted alongside harmful incident rates, exposure denominators, reporting volumes, and scores from validated safety culture instruments such as the Safety Attitudes Questionnaire [29] and the Hospital Survey on Patient Safety Culture [30]. Triangulation addresses the limitation of voluntary reporting systems, in which fluctuations may reflect cultural variation more than underlying risk [8].

### Ethical Considerations

This study did not involve human participants or patient-level data. All information was derived from peer-reviewed literature and publicly available institutional reports. Therefore, institutional review board approval was not required. Organizations implementing the framework with operational data should obtain local quality improvement or institutional review board determinations as appropriate.

## Results

The outcome of this study is a 3-level framework for decision-grade analysis of near miss data. The framework integrates taxonomy, operational definitions, SPC, and severity weighting.

### Framework Artifact

**Figure 1.**
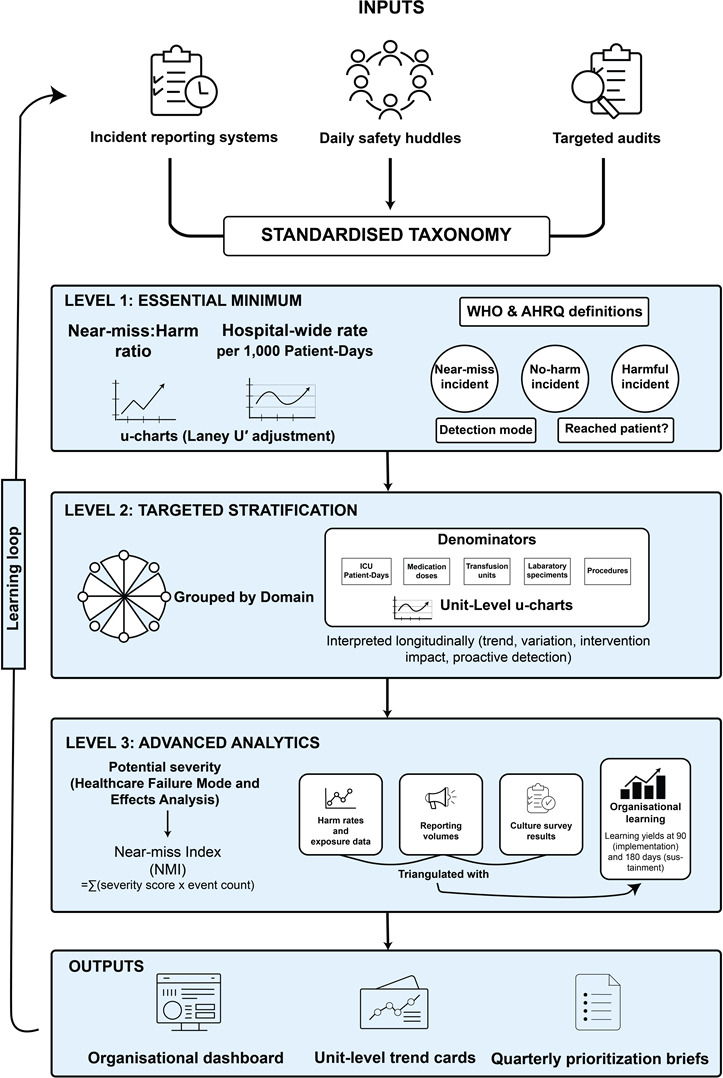
Framework architecture for decision-grade near miss analytics. Inputs from incident reporting systems, daily safety huddles, and targeted audits are standardized using World Health Organization (WHO) and Agency for Healthcare Research and Quality (AHRQ) definitions of near miss, no-harm incident, and harmful incident, together with event descriptors such as detection mode and whether the patient was reached. Data then progresses through 3 maturity levels. Level 1 (essential minimum) produces hospital-wide near miss rates per 1000 patient-days and near miss–to-harm ratios, monitored with u-charts (control charts for attributes) adjusted using the Laney U’ adjustment method when overdispersion is present. Level 2 (targeted stratification) applies domain-specific denominators (intensive care unit [ICU] patient-days, medication doses, laboratory specimens, transfusion units, and procedures) and generates unit-level u-charts that are interpreted longitudinally. Level 3 (advanced analytics) weights near misses by potential severity using the Institute for Healthcare Improvement Severity Assessment Code (IHI SAC) scale and calculates a Near Miss Index (NMI=Σ[severity score×event count]). Near miss data are triangulated with harm rates, exposure measures, reporting volumes, and safety culture survey scores and are linked to organizational learning yields at 90 and 180 days (action implementation and sustainment). Outputs include an organizational dashboard, unit-level trend cards, and quarterly prioritization briefs.

The framework begins with the capture of events through 3 complementary input channels: voluntary incident reporting systems, daily safety huddles, and targeted audits. In daily huddles, frontline staff verbally flag safety concerns and near miss events; a designated recorder enters qualifying events into the incident reporting system using the same WHO and AHRQ classification fields, ensuring a single integrated dataset. Audit-identified events, such as medication discrepancies found during pharmacy reconciliation, are likewise entered through the reporting system. Therefore, all inputs are coded using the WHO and AHRQ classifications to ensure consistency of terms. Data elements include event type, a patient-reached flag, detection mode, and unit or domain. The information is processed through successive levels. The first level produces organization-wide rates and ratios with SPC monitoring. The second level produces domain-specific rates using aligned denominators and unit-level charts. The third level weights events by severity, computes an NMI, and links reporting to corrective actions and culture data. The flow from inputs to outputs, with feedback to learning systems, is depicted in Figure 1.

### Interpretation Matrix

The framework also serves as a structured aid for leadership interpretation. Patterns in near miss reporting require context. In some instances, near misses increase while harm decreases. This suggests stronger interception and more open reporting. In other cases, near misses increase while total reports remain flat. This indicates better detection in selected domains. A third pattern is when both near misses and overall reports increase. This is more complex and may reflect cultural shifts and emerging system strain. A fourth scenario arises when both near misses and harm decrease simultaneously, which may signal declining reporting engagement rather than genuine safety improvement. A fifth scenario, in which near miss and harm rates remain stable with no SPC signals, may indicate system equilibrium but still warrants periodic review to guard against complacency. Each of these patterns is linked to diagnostic checks such as denominators, SPC variation, harm rates, and culture scores. The matrix is presented in Table 3. It structures trends into observed patterns, diagnostic considerations, and leadership implications.

**Table 3.** Interpretation matrix for leadership decision-making^a^.

Observed trend	Diagnostic checks	Possible interpretation	Leadership action
Increasing near misses while harm decreases or remains flat	Stability of denominators and SPC^b^ signals	Interception and reporting culture have improved	Reinforce effective defenses and promote open reporting
Increasing near misses while overall reporting remains stable	Clustering in specific domains and detection processes in those areas	Detection has improved in selected domains	Investigate process precursors and address system vulnerabilities
Both near misses and overall reports increase	Harm rates, exposure changes, system vulnerabilities, and SPC variation	Reporting culture is improving, but system stress may be rising	Review high-consequence clusters using the Near Miss Index, apply corrective actions, and monitor sustainment
Both near misses and harm decrease	Culture survey trends, reporting volume, and staff engagement data	Reporting fatigue or disengagement rather than true safety improvement	Conduct targeted culture assessment, reinforce nonpunitive reporting, and verify harm data completeness
Both near misses and harm remain flat (no SPC signals)	Denominator stability, process changes, and culture survey trends	The system is at equilibrium, or surveillance sensitivity has plateaued	Conduct periodic review of detection processes and consider proactive audits to test reporting sensitivity

a This matrix presents 5 common patterns in near miss reporting and links each pattern to diagnostic checks, possible interpretations, and recommended leadership actions. The aim is to provide consistent decision guidance and reduce the risk of misinterpreting reporting variation as changes in underlying risk.

b SPC: statistical process control.

### Application and Governance Outputs

A synthetic example was constructed to demonstrate the framework’s application, using parameters detailed in Multimedia Appendix 1 [19,23,27,28,31]. In a hypothetical quarter, 50 reported harmful incidents and 10 reported near misses were modeled across 50,000 patient-days. This yields a reported harm rate of 1.0 per 1000 patient-days and a near miss rate of 0.2 per 1000 patient-days. The resulting reported near miss-to-harm ratio is 0.2:1, reflecting the common underreporting of near misses. Severity was then assigned using a weighted scale (minor=1, moderate=2, major=3, and catastrophic=5). The 10 near misses were distributed as 5 minor, 3 moderate, 1 major, and 1 catastrophic, which computes a severity-weighted NMI of 19. Organizational response was modeled over time: at 90 days, 3 corrective actions were implemented for a learning yield of 30% (3/10); at 180 days, 2 of those actions were sustained for a sustainment rate of approximately 67% (2/3). This example illustrates how the NM³ framework converts raw and even sparse reported data into standardized rates, a risk-prioritized index, and actionable governance metrics. The detailed rationale for each parameter is provided in Multimedia Appendix 1.

The framework produces outputs that can be integrated into governance processes. The first level produces a monthly organizational dashboard with standardized rates and SPC signals. The second level provides unit-level reports with stratified denominators and longitudinal trends. The third level delivers a quarterly report with weighted indices, learning yields, and triangulated indicators. Together, these outputs move near miss reporting from descriptive tallies to decision-grade intelligence that supports prioritization and oversight.

## Discussion

### Principal Findings

This study developed a 3-level framework to strengthen the analysis of near miss data. The framework integrates taxonomy, rate calculation, SPC, severity weighting, and learning metrics. It addresses the current challenge that near miss reporting often produces data but not reliable interpretation. By combining definitional clarity with analytic structure, it converts descriptive data into decision-grade intelligence.

The contribution of this work lies in its ability to unify 3 methodological strands. The first is a standardized classification of events, which ensures that data reflect common definitions rather than local interpretation. The second is the application of SPC, which provides stable detection of variation over time and guards against overdispersion. The third is the use of severity weighting and learning metrics, which elevate high-consequence signals and link reporting to organizational action. Together, these strands close the gap between reporting and governance.

The NM³ framework can be understood as a decision support architecture within a health care sociotechnical system [15,34,35]. By standardizing how safety events are classified, stabilized over time, weighted by potential consequence, and interpreted in relation to exposure and culture metrics, the framework reduces cognitive ambiguity in governance decision-making. Rather than leaving interpretation to informal heuristics, NM³ provides a shared analytic structure that supports consistent situational awareness across organizational levels [36]. In this way, near miss analytics shift from a descriptive reporting exercise to a structured component of human-centered safety governance [37].

### Comparison With Prior Work

Existing approaches often remain descriptive. Most incident reporting systems provide tallies and categorical breakdowns but do not resolve the dilemma of what rising or falling near miss counts mean. The WHO ICPS established a definitional foundation [25], but it does not specify analytic processes. The structure-process-outcome model by Donabedian [38] underpins quality measurement broadly, but it does not address the specific problem of near miss interpretation. More recent frameworks, such as the Value Transformation Framework in primary care [39], focus on organizational change at the system level rather than the metrics of event reporting. Therefore, this study adds a new lens that sits between operational reporting and strategic governance.

The framework also supports practical adoption. Levels are designed as a maturity pathway. Organizations at baseline can implement level 1 by adopting a taxonomy and calculating standardized rates with simple u-charts. More advanced organizations can progress to level 2, where stratification by domain exposes hot spots without generating cross-unit competition. The most mature can reach level 3, where severity weighting, NMIs, and learning yields allow prioritization of risk and assessment of system responsiveness. In this way, the framework adapts to capacity rather than demanding wholesale transformation at once. In terms of scale, level 1 analytics can be applied by any hospital with a functioning incident reporting system. For level 2 stratification, sufficient denominator volume is needed to produce stable u-charts at the unit level; where monthly event counts are very low, such as in small community hospitals, a quarterly aggregation, as specified in the framework, preserves analytic validity. Level 3 severity weighting and learning yields are most informative when the organization reports at least several dozen near misses per quarter, providing enough events for meaningful index calculation and trend detection.

For governance, the framework strengthens the link between frontline reporting and board oversight. Dashboards at level 1 provide stable high-level indicators. Stratified reports at level 2 support managers who need to identify domain-specific risks. The advanced analytics of level 3 help leaders focus on high-consequence clusters and track whether actions are sustained. This aligns with international calls for safety metrics that are not only valid but actionable.

Future work must test and refine the framework. Empirical studies should evaluate its feasibility in practice, assess the reliability of severity scoring, and compare outputs across institutions. Research should also explore how digital systems can automate rate calculation, control chart production, and NMI computation. Benchmarking across hospitals and international contexts will determine generalizability and adaptation needs. Such validation will establish whether the framework produces consistent intelligence and supports decisions that improve safety outcomes. Furthermore, research should explore the integration of qualitative data from near miss narratives with the quantitative outputs of the NM³ framework to provide richer contextual understanding.

### Limitations

This framework has important limitations. It depends on voluntary reporting systems that are known to undercount events substantially; estimates suggest that voluntary systems capture as few as 5% to 10% of patient safety incidents, with near misses particularly underrepresented [7,9]. Reporting behavior is shaped by culture, workload, and fear, and these influences may distort numerators. Denominator data also present challenges. Accurate counts of patient-days, doses, or procedures are not always available or consistent across systems. Errors in denominators can lead to misleading rates.

Event classification introduces uncertainty at level 1: even with standardized WHO and AHRQ definitions, assigning the correct event type depends on individual reviewer judgment, and variability in interpretation may introduce classification error. At level 3, severity scoring presents a similar challenge; the IHI SAC score provides structure, but interrater reliability was not tested here. Training, calibration exercises, and periodic agreement checks are recommended for both event classification and severity scoring. Learning yield metrics depend on robust action tracking, which is not consistently available in many organizations.

Finally, this study is conceptual. The framework was derived from literature and policy rather than empirical testing. Its generalizability across different health care contexts remains uncertain until validation is undertaken. Pilot applications will be necessary to confirm feasibility, reliability, and value in governance. Implementation also presupposes dedicated human resources and analytic expertise. This may not be available, particularly for level 2 stratification and level 3 severity weighting in resource-constrained settings, and organizations should assess workforce readiness before progression beyond level 1.

### Implications

This framework has direct implications for health care organizations, quality leaders, and researchers. For organizations, the staged design lowers the barrier to adoption by allowing hospitals to begin with standardized baseline measurement and progress to more advanced analytics as institutional capacity grows, rather than requiring wholesale transformation at the outset.

For leadership, NM³ converts descriptive reporting into actionable intelligence that supports evidence-based governance. The tiered outputs provide a common language for safety oversight and enable boards to distinguish changes in reporting culture from genuine shifts in underlying risk, aligning with international calls for actionable patient safety metrics.

For researchers, NM³ provides a reproducible conceptual model that can be operationalized and validated across diverse health care contexts. Adoption in varied settings will help establish its role in patient safety improvement and determine the conditions under which the framework yields the greatest analytic value.

### Conclusions

This study developed a 3-level framework for decision-grade interpretation of near miss data within acute hospital systems. The framework integrates taxonomy, SPC, severity weighting, and organizational learning metrics. It addresses the ambiguity that has long limited the use of near miss reports in governance.

By structuring analysis into progressive levels, the framework functions as a cognitive and organizational scaffold for interpreting safety signals in complex sociotechnical environments. It enables leaders to distinguish culture shifts from genuine changes in risk and to prioritize high-consequence signals. Adoption has the potential to strengthen human-centered safety governance and support proactive risk mitigation.

## Supplementary material

10.2196/87846 Multimedia Appendix 1 Rationale for synthetic example parameters.

## Abbreviations

AHRQ: Agency for Healthcare Research and Quality
ICPS: International Classification for Patient Safety
IHI: Institute for Healthcare Improvement
NMI: Near Miss Index
NM³: 3-level near miss framework
SAC: Severity Assessment Code
SPC: statistical process control
WHO: World Health Organization
