# Supplementary material for: Near Miss Reporting and Organizational Learning in Health Care: Conceptual Framework Development Study
Source: JMIR Hum Factors. 2026 Apr 22;13:e87846. doi: 10.2196/87846 (PMC13102322; doi:10.2196/87846)
Supplement: Multimedia Appendix 1 [file humanfactors-v13-e87846-s001.docx]

**Supplement**

**Rationale for the Synthetic Example Parameters**

This appendix details the construction of the synthetic dataset used in the main manuscript’s illustrative application. The parameters were selected to model a realistic baseline that reflects paradoxical patterns in voluntary reporting data, where reported near misses are often significantly fewer than reported harms, contrary to theoretical expectations. The example is didactic, not empirical, and is designed to demonstrate the application of NM³ analytics to a common but suboptimal reporting profile.

**1. Definitions and Scope**

To ensure methodological clarity, incident classes are defined according to the World Health Organization (WHO) International Classification for Patient Safety (ICPS) and the Agency for Healthcare Research and Quality (AHRQ) Patient Safety Network (PSNet) ^1,2^.

- **Near miss:** An incident that was intercepted or did not reach the patient ^1^.
- **No-harm incident:** An incident that reached the patient but resulted in no discernible harm ^1^.
- **Harmful incident (Adverse Event):** An incident that resulted in harm to a patient ^2^.

**2. Exposure Denominator**

We set the exposure at **50,000 patient-days per quarter**. This value represents a plausible denominator for a large hospital, suitable for stable rate estimation. This order of magnitude is supported by national data from the U.S. Census Bureau Quarterly Services Survey, which reports approximately 57 million total inpatient days per quarter nationally ^3^.

**3. Reported Harms**

We model **50 reported harmful incidents** for the quarter, yielding a rate of **1.0 per 1,000 patient-days**. This rate is consistent with empirical data from hospital incident reporting systems. For instance, Nuckols et al. found that rates of reported events causing harm in established systems were often in the range of 1 to 3 per 1,000 patient-days, making our modeled rate conservative but consistent with reported ranges ^4^.

**4. Reported Near Misses and the Reported NM:H Ratio**

We model **10 reported near misses**, yielding a rate of **0.2 per 1,000 patient-days**. This creates a **Reported Near Miss-to-Harm (NM:H) Ratio of 0.2:1** (i.e., one reported near miss for every five reported harms). This ratio is deliberately chosen to illustrate the profound underreporting of near misses. For example, analysis of the CHPSO patient safety database, where near misses are ~11% of reports and harms are ~71%, equates to an approximate real-world NM:H ratio of 0.15:1 (11 ÷ 71) ^5^. Our modeled 0.2:1 ratio is therefore consistent with these observed bounds.

**5. Statistical Process Control (SPC)**

Healthcare attribute data, such as incident counts, frequently exhibit overdispersion (more variation than assumed by the Poisson distribution). Therefore, we specify the use of SPC **u-charts with Laney’s u′ modification** to adjust control limits, ensuring stable and valid signal detection ^6^.

**6. Severity Distribution and Near-Miss Index (NMI)**

We weight the potential consequence of the 10 near misses using the IHI/NPSF SAC framework four-band structure with numerical weights **(Minor=1, Moderate=2, Major=3, Catastrophic=5) ^7^**. We allocate the events as **5 minor, 3 moderate, 1 major, and 1 catastrophic** to reflect the typical right-skewed distribution of event severity. This computes a severity-weighted **NMI of 19**. This NMI value is not intended to represent a real-world NMI magnitude, only to illustrate the calculation method; its low absolute value is a direct consequence of the small number of reported near misses in this didactic example.

**7. Learning Yield and Sustainment Horizons**

Systematic reviews of incident reporting systems indicate that only a minority of reports lead to implemented corrective actions. For example, the systematic review by Goekcimen et al. found that among studies reporting such data, systemic changes were implemented in response to approximately 36% of incidents ^8^. Accordingly, we model a **Learning Yield of 30%**, where 3 of the 10 near misses result in an implemented action by 90 days. As empirical data on the long-term sustainment of such actions are scarce, we model a **sustainment rate of ~67%** (2 of 3 actions) at 180 days to illustrate the governance use of this crucial implementation outcome metric, consistent with Proctor’s taxonomy of implementation outcomes ^9^.

**Table A1. Basis for Synthetic Parameters**

| Analytic Element | Synthetic Value | Empirical or Methodological Anchor | Sources |
| --- | --- | --- | --- |
| Exposure Denominator | 50,000 patient-days per quarter | Plausible for a large hospital, supported by U.S. Census Bureau national aggregate data. | ^3^ |
| Reported Harms | 50 harms (1.0 / 1,000 patient-days) | Consistent with observed harm rates (1-3 per 1,000) in hospital IRS. | ^4^ |
| Reported Near Misses | 10 near misses (0.2 / 1,000 patient-days) | Reflects documented underreporting where reported NMs are few. | ^5^ |
| Reported NM:H Ratio* | 10 NM : 50 Harms = 0.2:1 | Modeled ratio is within observed bounds from large datasets (e.g., CHPSO ≈ 0.15:1). | ^5^ |
| SPC Method | u-chart with Laney u′ | Standard method to correct for overdispersion common in healthcare data. | ^6^ |
| Severity Distribution | 5 minor, 3 moderate, 1 major, 1 catastrophic | Reflects typical right-skewed distribution of event severity using the IHI SAC framework. | ^7^ |
| Near-Miss Index (NMI) | NMI = 19 (using 1,2,3,5 weights) | Illustrates method; low value reflects low reporting volume. | ^7^ |
| Learning Yield (90 d) | 3/10 = 30% implemented | Consistent with reviews showing actions are implemented for a minority of reports (~36%). | ^8^ |
| Sustainment (180 d) | 2/3 = ~67% sustained | Illustrative value for "maintenance," a key implementation outcome. | ^9^ |

** Ratios are shown for demonstration only; reporting systems are not suitable for benchmarking NM:H values across sites.*

**References**

1. Runciman W, Hibbert P, Thomson R, Van Der Schaaf T, Sherman H, Lewalle P. Towards an International Classification for Patient Safety: key concepts and terms. **Int J Qual Health Care**. 2009;21(1):18-26. doi:10.1093/intqhc/mzn057

2. UC Davis PSNet Editorial Team. Adverse Events, Near Misses, and Errors. Rockville (MD): Agency for Healthcare Research and Quality, US Department of Health and Human Services, 2019. Accessed 11/07/2025, <https://psnet.ahrq.gov/primer/adverse-events-near-misses-and-errors>

3. U.S. Census Bureau. Total Inpatient Days for Hospitals, All Establishments [INPAT622ALLEST176QNSA],. Federal Reserve Bank of St. Louis, 2025. Accessed 25/07/2025, <https://fred.stlouisfed.org/series/INPAT622ALLEST176QNSA>

4. Nuckols TK, Bell DS, Liu H, Paddock SM, Hilborne LH. Rates and types of events reported to established incident reporting systems in two US hospitals. **Quality and Safety in Health Care**. 2007;16(3):164. doi:10.1136/qshc.2006.019901

5. Hospital Quality Institute. A Window into Patient Safety: Underreporting of Near-Miss Events in CHPSOData. 2024. Accessed 20/07/2025, <https://hqinstitute.org/a-window-into-patient-safety-underreporting-of-near-miss-events-in-chpsodata/>

6. Mohammed MA, Laney D. Overdispersion in health care performance data: Laney’s approach. **Quality and Safety in Health Care**. 2006;15(5):383. doi:10.1136/qshc.2006.017830

7. National Patient Safety Foundation. RCA2: Improving Root Cause Analyses and Actions to Prevent Harm. 2015. Accessed 18/07/2025, <https://www.ihi.org/library/tools/rca2-improving-root-cause-analyses-and-actions-prevent-harm>

8. Goekcimen K, Schwendimann R, Pfeiffer Y, Mohr G, Jaeger C, Mueller S. Addressing Patient Safety Hazards Using Critical Incident Reporting in Hospitals: A Systematic Review. **Journal of Patient Safety**. 2023;19(1)doi:10.1097/PTS.0000000000001072

9. Proctor E, Silmere H, Raghavan R, et al. Outcomes for Implementation Research: Conceptual Distinctions, Measurement Challenges, and Research Agenda. **Administration and Policy in Mental Health and Mental Health Services Research**. 2011;38(2):65-76. doi:10.1007/s10488-010-0319-7
